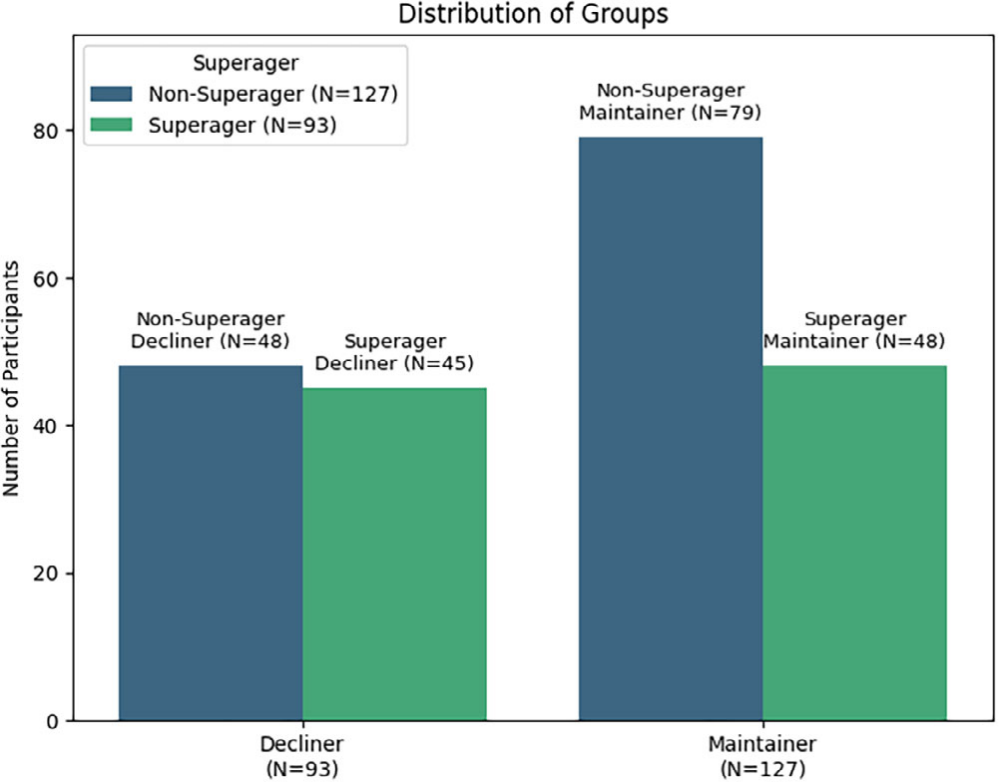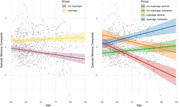# Exploring Memory Trajectories in Aging: A Longitudinal Study of Superagers and Memory Maintenance

**DOI:** 10.1002/alz70857_102745

**Published:** 2025-12-25

**Authors:** Rachel M Morse, Cristina Solé‐Padullés, María Cabello‐Toscano, Lídia Mulet‐Pons, Oriol Perera‐Cruz, Javier Solana Sánchez, Gabriele Cattaneo, Alvaro Pascual‐Leone, David Bartrés‐Faz

**Affiliations:** ^1^ Department of Medicine, Faculty of Medicine and Health Sciences, Institute of Neurosciences, University of Barcelona, Barcelona, Spain; ^2^ Institute of Biomedical Research August Pi i Sunyer (IDIBAPS), Barcelona, Spain; ^3^ Department of Medicine, Faculty of Medicine and Health Sciences and Institute of Neurosciences, University of Barcelona, Barcelona, Barcelona, Spain; ^4^ August Pi I Sunyer Institute of Biomedical Research (IDIBAPS), Barcelona, Barcelona, Spain; ^5^ Department of Medicine, Faculty of Medicine and Health Sciences, Institute of Neurosciences, University of Barcelona, Barcelona, 08036, Spain; ^6^ Institut Guttmann, Institut Universitari de Neurorehabilitació adscrit a la Universitat Autònoma de Barcelona, Badalona, Barcelona, Spain; ^7^ Fundació Institut d’Investigació en Ciències de la Salut Germans Trias i Pujol, Badalona, Barcelona, Spain; ^8^ Universitat Autònoma de Barcelona, Bellaterra (Cerdanyola del Vallès), Spain; ^9^ Fundació Institut d'Investigació en Ciències de la Salut Germans Trias i Pujol, Badalona, Spain; ^10^ Hinda and Arthur Marcus Institute for Aging Research and Deanne and Sidney Wolk Center for Memory Health, Hebrew SeniorLife, Department of Neurology, Harvard Medical School, Boston, MA, USA; ^11^ Department of Neurology, Harvard Medical School, Boston, MA, USA

## Abstract

**Background:**

While many individuals’ episodic memory declines after age 60, this decline is not universal. Some individuals, often called superagers, have exceptional memory in older age. Despite growing research interest in superagers, their long‐term memory trajectories remain underexplored.

**Method:**

We used longitudinal data from 220 participants (127 female, age_mean_=67.3 years, follow‐up_mean_=2.2 years) from the Barcelona Brain Health Initiative and the University of Barcelona Aging cohorts. We calculated episodic memory composites using the Face‐Name Associative Memory Test and the Rey Auditory Verbal Learning Test (RAVLT), and memory change using ordinary least squares slope estimates. We classified participants as ‘superagers’ if they scored at/above the mean for 16–29‐year‐olds on the RAVLT delayed recall and above 1SD below the mean for their age/education on the Trail Making Test B. We classified participants as ‘maintainers’ if their memory change was >=0. We then created four groups: superager maintainers, superager decliners, non‐superager maintainers, and non‐superager decliners. We conducted ANOVAs to compare groups’ memory at baseline/follow‐up and linear mixed effects models with a group/age interaction on memory. We controlled analyses for relevant demographics.

**Result:**

Figure 1 summarizes participant distribution across groups. Superagers had significantly better memory than non‐superagers at baseline/follow‐up (*p*s<0.001). Initially, decliners exhibited better memory than maintainers (*p* < 0.001), but this trend reversed at follow‐up with maintainers showing better memory (*p* < 0.001). Superagers, compared to non‐superagers (β=0.041, *p* = 0.005), and maintainers, compared to decliners (β=0.136, *p* < 0.001) showed positive interactions with age on memory. Similarly, compared to non‐superager decliners, superager maintainers (β=0.147, *p* < 0.001) and non‐superager maintainers (β=0.108, *p* < 0.001) exhibited significant positive interactions with age on memory, unlike superager decliners (β=0.042, *p* = 0.079) (Figure 2).

**Conclusion:**

Superager status benefited memory level and memory change while maintainer status benefited memory change and memory level at follow‐up only. Superagers can be further categorized into superager maintainers, who exhibit a more positive relationship between memory and age than superager decliners. Similarly, non‐superager maintainers show a more positive relationship between memory and age than non‐superager decliners. These findings underscore the complexity of cognitive aging trajectories, indicating superager status may offer insights into longitudinal patterns, though additional subgroups further clarify these relationships.